# A Descriptive Analysis of Potential Warfarin-NSAID Interactions in Dental Prescribing in Minas Gerais, Brazil, 2011–2021

**DOI:** 10.3390/healthcare14101326

**Published:** 2026-05-13

**Authors:** Jennifer Reis-Oliveira, Alex Junio Silva da Cruz, Widla Emanuella Pereira Barreto Garcez, Jacqueline Silva Santos, Maria Auxiliadora Parreiras Martins, Mauro Henrique Nogueira Guimarães de Abreu

**Affiliations:** 1 Department of Community and Preventive Dentistry, School of Dentistry, Universidade Federal de Minas Gerais, Belo Horizonte 31270-800, MG, Brazil; jenniferreisoliveira@ufmg.br (J.R.-O.); aame0590@ufmg.br (A.J.S.d.C.); widla@ufmg.br (W.E.P.B.G.); jacqueline.silva@saude.mg.gov.br (J.S.S.); 2 Department of Pharmaceutical Products, School of Pharmacy, Universidade Federal de Minas Gerais, Belo Horizonte 31270-800, MG, Brazil; auxiliadorapmartins@ufmg.br

**Keywords:** warfarin, anti-inflammatory agents, non-steroidal, drug interactions, dental care, patient safety

## Abstract

Background/Objectives: The use of nonsteroidal anti-inflammatory drugs (NSAIDs) is common in dentistry, mainly for pain and inflammation. However, their coadministration with warfarin may lead to serious potential drug-drug interactions (PDDIs), increasing the risk of bleeding. This study aimed to identify and describe the frequency of PDDIs between warfarin and NSAIDs prescribed by dentists and dispensed by the Unified Health System (SUS) in Minas Gerais, Brazil, from January 2011 to December 2021. Methods: A descriptive analysis was conducted using data from Integrated Pharmaceutical Services Management System (Sigaf), considering prescriptions of warfarin and NSAIDs issued during the same period. Results: The prescribed NSAIDs were diclofenac sodium 50 mg, diclofenac potassium 50 mg, ibuprofen 600 mg, nimesulide 100 mg, and nimesulide 50 mg/mL oral suspension. Warfarin sodium 5 mg is the prescribed oral anticoagulants. The results showed a marked increase in both warfarin (from 6017 to 59,945 prescriptions; +896%) and NSAID use (from 2644 to 84,408 prescriptions; +3093%), paralleling the rise in PDDIs, which grew from 2 in 2011 to 62 in 2021. Despite this 3000% relative increase, the absolute frequency of PDDIs remained low, corresponding to approximately 0.7 interactions per 1000 NSAID prescriptions in 2021. Conclusions: Although these PDDIs are low, they are clinically significant and may have important implications for patients and the healthcare system. In conclusion, PDDIs between NSAIDs and warfarin, though low in absolute numbers, have increased over the years, reinforcing the need for greater awareness among dental professionals and for the implementation of clinical decision support strategies to promote safe care.

## 1. Introduction

Nonsteroidal anti-inflammatory drugs (NSAIDs) are widely used in healthcare because of their anti-inflammatory, antipyretic (fever-reducing), and analgesic (pain-relieving) properties [1]. They primarily act by inhibiting cyclooxygenase enzymes (COX-1 and COX-2), thereby reducing the synthesis of prostaglandins that mediate pain and inflammation [2]. Traditionally classified according to their chemical characteristics and selectivity, examples include propionic acids (naproxen, ibuprofen), acetylated salicylates (aspirin), acetic acids (diclofenac, indomethacin), anthranilic acids (meclofenamate, mefenamic acid), non-acetylated salicylates (diflunisal, salsalate), naphthylalanines (nabumetone), oxicams (piroxicam, meloxicam), and selective COX-2 inhibitors (celecoxib, etoricoxib) [3].

In dentistry, NSAIDs are extensively used for pain and inflammation control and are considered the first-line medications for endodontic, surgical, and periodontal procedures [4,5]. Although other medications, such as opioid analgesics, are available for pain management, the American Dental Association (ADA) guidelines state that NSAIDs are not only safer than opioids but also more effective [6]. Furthermore, recent epidemiological and cross-sectional studies show a shift in prescribing patterns in North America, with a relative increase in non-opioid analgesic prescriptions and changes in the proportion of dental prescriptions within the broader prescribing context. These trends reflect adherence to contemporary guidelines and efforts to curb opioid use [7,8,9].

In Brazil, there has been a significant increase in NSAID prescriptions in dentistry [10]. Ibuprofen, nimesulide, and diclofenac are among the most prescribed and consumed NSAIDs in dental settings, underscoring the influence of over-the-counter availability on usage patterns and highlighting the need for guidance on rational use and adverse event monitoring [11,12].

Potential drug-drug interactions (PDDIs) occur when one drug alters the effect of another, either increasing or decreasing its therapeutic action. Recognizing and managing these interactions is fundamental in clinical practice, as inadequate monitoring may lead to serious outcomes, such as unexpected toxicity or therapeutic failure. Therefore, surveillance contributes to the early identification of clinical manifestations of PDDIs and their management with the aim of promoting patient safety [2,13]. With the rise of polypharmacy, particularly among older adults and patients with multiple comorbidities, the risk of PDDIs increases, requiring careful and individualized assessment to ensure treatment safety and efficacy [14].

The prescription of NSAIDs requires a thorough assessment of the patient’s clinical profile, given the wide range of PDDIs that may compromise therapeutic safety and effectiveness. Inhibition of prostaglandin synthesis by NSAIDs can antagonize the effects of antihypertensives, resulting in elevated blood pressure and an increased risk of acute kidney injury, particularly in older individuals or those with heart failure [2,15]. Other clinically significant PDDIs include concomitant use with lithium or methotrexate, as the reduction in renal clearance of these drugs can lead to accumulation and systemic toxicity [16,17]. The primary concern, however, involves the concomitant use of NSAIDs with oral anticoagulants, such as warfarin, because inhibition of platelet aggregation combined with potential gastric mucosal irritation may increase the risk of severe bleeding, such as hemorrhagic stroke [15,18,19]. Consequently, patients are at risk of developing serious medication-related events.

Despite the clinical relevance of PDDIs, studies indicate that dentists often demonstrate limited knowledge on this topic, which may compromise the safety of dental care. Evidence from studies conducted in India, Lebanon, and Brazil corroborates this finding [20,21,22]. Given the limited knowledge among dental professionals regarding PDDIs, coupled with the increasing number of patients using multiple prescribed medications [23], early detection of PDDIs is essential for providing appropriate guidance that can enhance patient safety. Additionally, Garcez et al. (2025) [24] reported that the prevalence of PDDIs between warfarin and NSAIDs, based on prescriptions analyzed in the state of Minas Gerais in 2021, was 0.41%. However, longitudinal studies are not yet available in the literature, despite their importance for surveillance and public health planning. Therefore, this study aimed to describe the NSAIDs prescribed by dentists and dispensed through the Unified Health System (SUS) in Minas Gerais, southeastern Brazil, between 2011 and 2021, and to determine the differences in the prevalence of PDDIs involving warfarin and NSAIDs in the state.

## 2. Materials and Methods

### 2.1. Ethical Considerations

The present study was approved by the Research Ethics Committee of the Universidade Federal de Minas Gerais (UFMG), registered under protocol number CAAE 88465118.8.0000.5149; approval date: 27 August 2024. Since this study used secondary data routinely collected by health services, the requirement to obtain Informed Consent Forms from patients and prescribers was waived.

### 2.2. Study Population and Data Source

A descriptive analysis was conducted using the Integrated Pharmaceutical Services Management System (Sigaf) database for the state of Minas Gerais, Brazil, covering the period from January 2011 to December 2021. This statewide database has been implemented in all 853 municipalities of Minas Gerais since 2009. In addition to supporting pharmaceutical management in public pharmacies, the system includes inventory records, medication dispensing history, quantity, year, dosage, identification of the prescribing health professional, and patient identifiers. This period, 2011 to 2021, was chosen because it allows for the capture of long-term trends in a low-frequency outcome (PDDIs) and because it coincides with the consolidation and expansion phase of Sigaf coverage in the state, enabling a more robust analysis of the phenomenon’s evolution.

**Figure 1:**
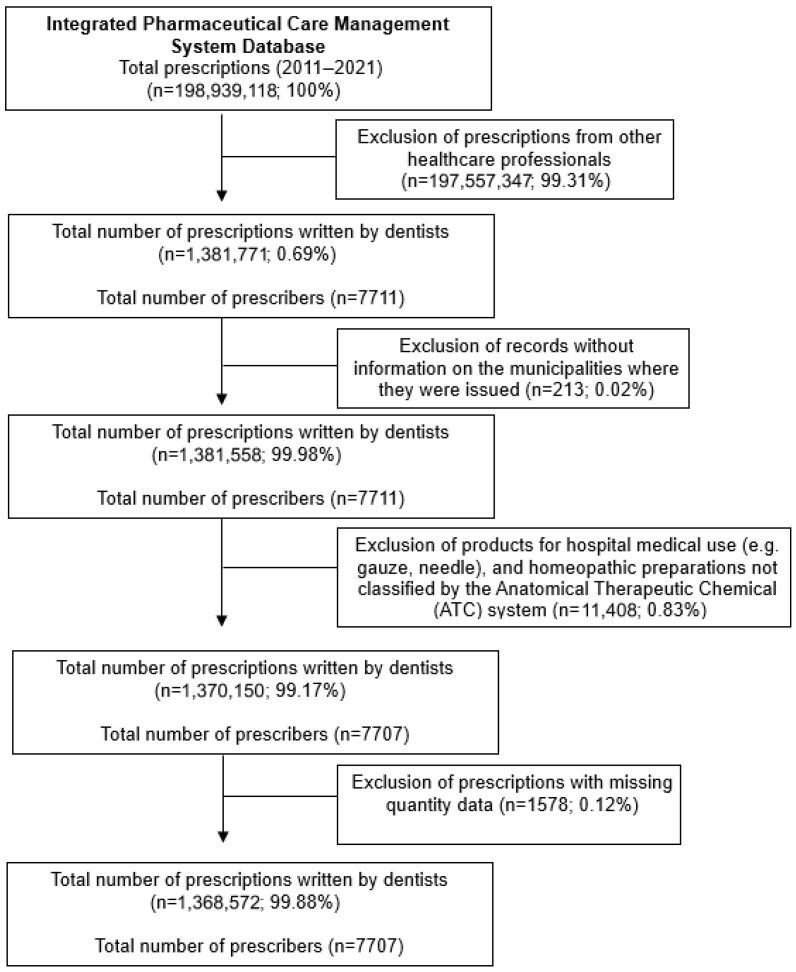
Selection of dental prescriptions (NSAIDs).

In the first stage of the study, all individuals who received a warfarin prescription recorded in the Sigaf database were selected, stratified by year and municipality. Individuals residing outside Minas Gerais, those with missing data on age and/or sex, and/or those under 18 years of age were excluded from analysis. In the subsequent phase, individuals who received a dental prescription for an NSAID within the same database were identified (Figure 1).

To evaluate the concomitant use of these medications, the Defined Daily Dose (DDD) methodology was applied, as defined by the World Health Organization. The DDD represents the assumed average maintenance dose per day of a drug used for its main indication in adults [25,26]. The number of DDDs is calculated by multiplying the number of units dispensed by the dosage in milligrams (mg) and dividing the result by the drug’s specific DDD [27,28].

To illustrate the process, consider a prescription of eight 600 mg ibuprofen tablets, starting on 1 November 2020. The WHO-defined DDD for ibuprofen is 1200 mg. The total amount of active ingredient dispensed would be calculated as follows: 8 × 600 mg = 4800 mg. Dividing this total by the DDD (1200 mg) results in 4 DDDs. Thus, according to the WHO-established DDD, the prescribed quantity would correspond to four days of treatment. Assuming initiation on 1 November 2020, the exposure period would extend until 4 November 2020.

To determine concomitant exposure, the dispensing date was considered the first day of treatment. The duration of treatment for each prescription was estimated by dividing the total amount of the dispensed active ingredient by the drug’s DDD. Temporal overlap (PDDI) was identified when the estimated treatment period for an NSAID and for warfarin in the same patient coincided by at least one day. In cases of multiple prescriptions, each pair of medications was treated as an independent exposure episode. This is a standardized methodology widely used in drug utilization studies with secondary databases [24], allowing the identification of PDDIs from dispensing records, even though it does not reflect the patient’s exact adherence.

### 2.3. Study Variables and Data Analysis

A descriptive analysis was conducted covering the period from 2011 to 2021. For each year, the following were quantified: (i) The total number of prescriptions and patients using warfarin; (ii) The number of municipalities with recorded warfarin use; (iii) The total number of prescriptions and patients using NSAIDs; and (iv) The number of municipalities with recorded NSAIDs use. Additionally, patients with concomitant prescriptions of warfarin and NSAIDs were identified, as well as the municipalities where this co-occurrence took place. Based on this information, the annual number of PDDIs between warfarin and NSAIDs (the study outcome) was calculated, along with the number of municipalities in which these PDDIs were recorded.

Data were tabulated, allowing for the assessment of the history of warfarin use, NSAIDs use, and the occurrence of PDDIs throughout the study period. The statistical analysis was descriptive, presenting absolute values for each variable.

## 3. Results

Between 2011 and 2021, a substantial increase in warfarin use was observed (Table 1). The annual number of prescriptions rose from 6017 to 59,945, representing a change of approximately 896% during the period. The number of patients using the medication rose from 1250 to 14,539 (1063% increase). Regarding territorial coverage, the number of municipalities with records of warfarin use expanded from 127 in 2011 to 679 in 2021, an increase of 434%.

The growth trend for NSAIDs was even more pronounced. Yearly prescriptions jumped from 2644 in 2011 to 84,408 in 2021, representing an expansion of 3093%. The number of patients grew from 2147 to 68,030 over the same period (3068% increase), while municipalities with recorded use increased from 82 to 694 (746% increase). The year 2019 represented an inflection point, with an abrupt increase in NSAID prescriptions compared to previous years, suggesting an intensification of their use from that year onward.

The PDDIs between warfarin and NSAIDs, considered the main outcome of the study, also showed a substantial increase. In 2011, four patients had concomitant prescriptions, whereas in 2021, 122 patients were registered (2950% increase). The number of PDDIs grew from two in 2011 to 62 in 2021 (3000% change). Regarding territorial distribution, only one municipality recorded PDDIs in 2011, compared with 45 in 2021 (4400% increase). However, when standardized by the total volume of NSAID prescriptions, the rate of PDDIs remained consistently low and relatively stable throughout the study period, ranging from 0.2 to 0.9 interactions per 1000 prescriptions. Likewise, the proportion of warfarin users with a recorded interaction never exceeded 0.5%, reaching 0.41% in 2021 (Table 2).

**Figure 2:**
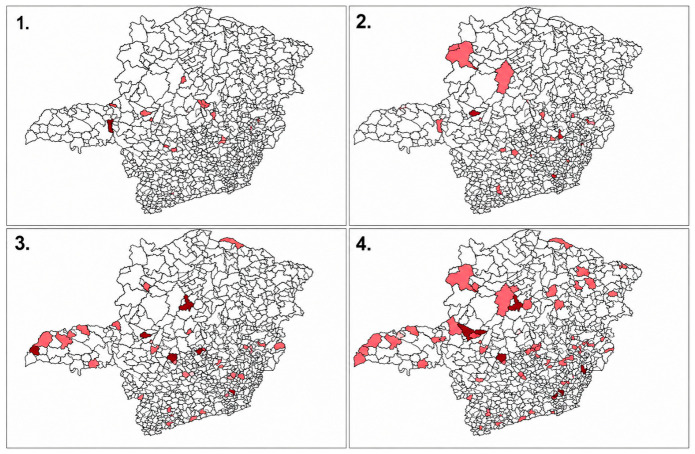
Geographic distribution of potential drug-drug interactions (PDDIs) between NSAIDs and warfarin in Minas Gerais, Brazil. The panel represents the periods 2011–2013 (Number 1), 2014–2016 (Number 2), 2017–2019 (Number 3), and 2020–2021 (Number 4). Light red indicates municipalities with one PDDI record, and dark red indicates municipalities with two or more PDDI records during the respective period.

Table 3 shows the list and geographic distribution of municipalities in Minas Gerais that registered PDDIs between NSAIDs and warfarin. Furthermore, the spatiotemporal expansion of these interactions across the state is visually represented in Figure 2.

## 4. Discussion

The results of this study indicate that PDDI between warfarin and NSAIDs remained relatively infrequent but increased steadily over the years. Despite their low absolute frequency, the severity of these PDDIs remains clinically relevant, as even isolated episodes may result in serious clinical outcomes, such as severe bleeding (especially gastrointestinal bleeding), stroke, or systemic embolism [19,28]. Therefore, the upward trend in PDDI should be interpreted as a warning signal for healthcare services and professionals involved in pharmacotherapeutic care.

The observed increase in PDDIs may be decomposed into two non-mutually exclusive phenomena. On the one hand, part of this growth likely reflects an enhanced capacity for surveillance and recording. Sigaf is a relatively recent system, and its consolidation over the last decade may have progressively improved the capture of prescriptions in Minas Gerais. The expansion of the Family Health Strategy (ESF) during the study period may also have increased healthcare coverage and, consequently, the likelihood of registering concomitant prescriptions in the database [29]. On the other hand, a genuine rise in co-exposure cannot be ruled out. The expansion of pharmaceutical assistance policies in Brazil, which provide both warfarin and several NSAIDs free of charge, has broadened population access to these medications [30]. Furthermore, secular changes such as population growth, demographic transition with an aging population, and the consequent increase in the prevalence of conditions requiring anticoagulation, as well as potential shifts in dental prescribing patterns over the decade, may have contributed to a real increase in the number of patients simultaneously exposed to both drug classes. Our descriptive design does not allow these factors to be disentangled, but by enumerating them, we provide hypotheses for future analytical investigations.

It is noteworthy that this increase occurred during a period when new oral anticoagulants were introduced to the market and gradually changed the landscape of anticoagulation in many settings, especially in the private healthcare system [19]. Despite this change, SIGAF data indicate that warfarin remained the predominant anticoagulant within the SUS in Minas Gerais, with its prescriptions growing steadily. This reinforces the relevance of the NSAID-warfarin PDDI as a current public health concern; had the use of warfarin been supplanted as in other healthcare systems, the absolute frequency of these PDDIs would likely be lower.

From a clinical and public health perspective, PDDIs involving warfarin and NSAIDs represent a relevant concern. Pharmacologically, the interaction results from the inhibition of platelet aggregation and gastrointestinal mucosal injury caused by NSAIDs, combined with the anticoagulant effect of warfarin, which inhibits the synthesis of vitamin K-dependent clotting factors [2,31]. This synergistic mechanism substantially increases the risk of severe bleeding. In the United States, the concomitant use of these drugs has been associated with a significantly elevated risk of hospitalization (HR: 1.64; 95% CI: 1.51–1.77; *p* < 0.0001), with costs ranging from US $10,000 to US $15,000 per hemorrhagic event [31,32,33,34]. Although prevalence is low, the clinical and socioeconomic impact of these events is disproportionately high, as they lead to hospitalizations, burden health services, and impair patients’ quality of life [35]. While these cost estimates are derived from a different healthcare context, they underscore the potential economic consequences that preventable drug interactions may impose on any health system, including the SUS.

In this context, advancing preventive strategies becomes essential. Targeted guidance for dentists, physicians, and pharmacists is crucial to reinforce awareness of the risks associated with this PDDI and to promote the rational prescription of analgesics in anticoagulated patients [2,21,35,36]. International evidence suggests that multiprofessional interventions and integrated clinical protocols may reduce the occurrence of PDDIs and improve patient safety [14,37].

It is important to note that although this study identified a growing trend in PDDIs during the period analyzed, it was not possible to conduct a time-series analysis. This limitation stems from the descriptive nature of the data, the absence of longitudinal information on therapeutic adherence, and the inability to establish causal relationships between prescribing trends and the occurrence of PDDIs. Therefore, the findings should be interpreted with caution and considered hypothesis-generating for future analytical investigations.

Among the study’s limitations, in addition to the inability to infer causality, is the exclusive reliance on administrative records from Sigaf, which may be subject to underreporting or inconsistencies. Moreover, it was not possible to assess clinical outcomes arising from PDDIs, such as hospitalizations or deaths, which restricts the evaluation of the actual impact on population morbidity and mortality. Additionally, the use of DDD as a proxy for treatment duration may not accurately reflect real-world medication use. Variations in prescribed doses, patient adherence, and short-term NSAID use may result in misclassification of concomitant exposure. Nevertheless, this study contributes by providing novel evidence on the occurrence of PDDIs in a dental care context in Brazil, underscoring the need for greater vigilance.

For future perspectives, retrospective cohort studies using secondary databases are needed to assess the direct clinical outcomes of these PDDIs, along with the promotion of educational interventions for healthcare professionals on the rational use of NSAIDs in anticoagulated patients. Furthermore, it is necessary to strengthen the dissemination of information about PDDIs in the dentist’s undergraduate education and on-the-job training, as well as collaboration with the multidisciplinary team. Qualitative and mixed-methods research should also be encouraged to investigate the pharmacology content taught in undergraduate dental curricula in Brazil, specifically assessing whether and how the warfarin-NSAID PDDIs are addressed. Understanding potential gaps in dental education regarding PDDIs may help explain prescribing behaviors and inform targeted curricular improvements. Additionally, the dispensing stage warrants investigation, as pharmacists represent the final safety barrier. The fact that these PDDIs materialized suggests potential failures at this juncture, such as the absence of integrated alert systems, ‘alert fatigue’ causing warnings to be overridden, or patients obtaining medications from different health units without a unified record. It is recommended that clinical decision support tools be developed and integrated directly into existing health information systems, such as Sigaf, so that they can issue electronic alerts at the time of prescribing or dispensing NSAIDs for patients with an active record of warfarin use, thereby prompting an active review of the prescription and mitigating this preventable risk [15].

## 5. Conclusions

In conclusion, PDDIs between warfarin and NSAIDs prescribed by dentists in the Minas Gerais SUS were infrequent, but their frequency increased over the study period. Despite their low prevalence, these PDDIs are clinically relevant and warrant greater attention from healthcare professionals, as well as strengthened prevention strategies, including clinical decision support tools, within the public health system.

## Figures and Tables

**Table 1:** General descriptive table of prescriptions, municipalities and PDDI * between NSAIDs ** and warfarin between 2011 and 2021 in the state of Minas Gerais.

	Warfarin Prescriptions	Warfarin Patients	*n* Municipalities Warfarin	NSAID Prescriptions	NSAID Patients	*n* Municipalities NSAIDs	Patients Prescribed NSAIDs and Warfarin	*n* Municipalities (Warfarin and NSAIDs)	Patients with PDDI	Total PDDI	*n* Municipalities with PDDI Warfarin and NSAIDs
**2011**	6017	1250	127	2644	2147	82	4	2	2	2	1
**2012**	19,951	2819	210	5963	4914	169	9	9	4	5	4
**2013**	26,366	3721	269	10,947	7986	195	18	16	10	10	10
**2014**	33,649	5781	426	13,222	10,748	268	14	5	5	7	5
**2015**	42,484	6969	464	20,367	16,467	328	33	11	13	16	11
**2016**	39,334	8132	456	28,528	23,105	391	33	6	8	9	6
**2017**	38,370	9437	498	39,213	26,066	420	40	5	6	6	5
**2018**	48,388	11,955	588	43,546	35,024	576	56	14	14	16	14
**2019**	56,435	14,135	654	72,269	57,189	687	114	26	30	32	26
**2020**	57,827	14,649	666	62,185	51,585	676	81	21	27	29	21
**2021**	59,945	14,539	679	84,408	68,030	694	122	45	60	62	45

* PDDI: potential drug-drug interactions. ** NSAIDs: nonsteroidal anti-inflammatory drugs.

**Table 2:** Annual rates of potential drug-drug interactions (PDDIs) between NSAIDs and warfarin standardized by prescription volume and by exposed population, Minas Gerais, Brazil (2011–2021).

Year	Total PDDIs	PDDIs per 1000 NSAID Prescriptions	Proportion of Warfarin Users with PDDI (%)
2011	2	0.8	0.16
2012	5	0.8	0.14
2013	10	0.9	0.27
2014	7	0.5	0.09
2015	16	0.8	0.19
2016	9	0.3	0.10
2017	6	0.2	0.06
2018	16	0.4	0.12
2019	32	0.4	0.21
2020	29	0.5	0.18
2021	62	0.7	0.41

**Table 3:** List of municipalities in Minas Gerais that recorded potential drug-drug interactions (PDDIs) between NSAIDs and warfarin (2011–2021).

Period	Number of Municipalities with PDDI per Year	Municipalities
2011–2013	2011: 1 2012: 4 2013: 10	Alpercata, Congonhas do Norte, Douradoquara, Guimarânia, Itumirim, Lagoa da Prata, Lagoa Formosa, Monjolos, Nova Ponte, Pedra do Indaiá, Pirapora, São Gonçalo do Rio Abaixo, São Sebastião do Rio Preto, Santa Rita de Minas
2014–2016	2014: 5 2015: 11 2016: 6	Alpercata, Aracitaba, Arapuá, Araporã, Belo Vale, Buritizeiro, Congonhas do Norte, Cláudio, Dom Bosco, Fortuna de Minas, Ibertioga, Lagoa Formosa, Lambari, Nova Era, Nova Ponte, Pedra do Indaiá, Rio Pomba, São Gonçalo do Rio Abaixo, Santa Rita de Minas, Unaí
2017–2019	2017: 5 2018: 14 2019: 26	Abadia dos Dourados, Ataleia, Barão de Cocais, Belo Oriente, Bom Despacho, Canápolis, Cláudio, Cristais, Dom Bosco, Douradoquara, Espinosa, Ibertioga, Itajubá, Itanhomi, Ituiutaba, Jequeri, Lagoa Formosa, Lambari, Limeira do Oeste, Mendes Pimentel, Morro da Garça, Muzambinho, Pará de Minas, Ponte Nova, Prudente de Morais, Resplendor, Rochedo de Minas, Santa Vitória, São José da Lapa, São José da Varginha, Senador Cortes, Serra Azul de Minas, Sete Lagoas, Tocos do Moji, Tupaciguara, Ubá, Várzea da Palma, Wenceslau Braz
2020–2021	2020: 21 2021: 45	Águas Formosas, Araújos, Arapuá, Baldim, Bandeira, Barão de Cocais, Belo Oriente, Bocaina de Minas, Bom Despacho, Bom Jardim de Minas, Buritizeiro, Cambuí, Capitólio, Carneirinho, Catas Altas, Conceição das Alagoas, Coromandel, Fernandes Tourinho, Francisco Dumont, Funilândia, Gonzaga, Iapu, Ibiaí, Indianópolis, Itacambira, Itajubá, Itaguara, Itanhomi, Itinga, Itueta, Jequitibá, Lagoa Formosa, Lagoa Santa, Limeira do Oeste, Manhuaçu, Marliéria, Muzambinho, Olímpio Noronha, Patos de Minas, Piracema, Prudente de Morais, Rio Pomba, Romaria, Salinas, Santa Vitória, São Domingos do Prata, São Geraldo do Baixio, São Gotardo, São Miguel do Anta, Sete Lagoas, Silvianópolis, Simão Pereira, Turvolândia, Ubá, Várzea da Palma, Viçosa

## Data Availability

The study data are not available for replication to protect patient confidentiality.

## Abbreviations

The following abbreviations are used in this manuscript:

ADA	American Dental Association
COX-1 and COX-2	Cyclooxygenase enzymes 1 and 2
DDD	Defined Daily Dose
ESF	Family Health Strategy
NSAIDs	Nonsteroidal anti-inflammatory drugs
PDDIs	Potential drug-drug interactions
Sigaf	Integrated Pharmaceutical Services Management System
SUS	Unified Health System
